# Comparative expression analysis identifies the respiratory transition-related miRNAs and their target genes in tissues of metamorphosing Chinese giant salamander (*Andrias davidianus*)

**DOI:** 10.1186/s12864-018-4662-5

**Published:** 2018-05-29

**Authors:** Shengyan Su, Yuheng Wang, Huiwei Wang, Wei Huang, Jun Chen, Jun Xing, Pao Xu, Xinhua Yuan, Caiji Huang, Yulin Zhou

**Affiliations:** 1 Key Laboratory of Genetic Breeding and Aquaculture Biology of Freshwater Fishes, Ministry of Agriculture; Freshwater Fisheries Research Center, Chinese Academy of Fishery Sciences, Wuxi, 214081 People’s Republic of China; 2 Department of Animal Husbandry & Veterinary Medicine, Jiangsu Polytechnic College of Agriculture and Forestry, Zhenjiang, 212400 People’s Republic of China; 3 Wuxi Fisheries College, Nanjing Agricultural University, Wuxi, 214081 People’s Republic of China

**Keywords:** Deep sequencing, Respiratory system, miRNA, *Andrias davidianus*

## Abstract

**Background:**

Chinese giant salamander (*Andrias davidianus*) undergoes a metamorphosis from aquatic larvae to terrestrial adults, with concomitant transfer of respiration from gills to lungs prior to metamorphosis. These two tissues, as well as skin, were sampled to identify the differentially expressed miRNAs.

**Results:**

High-coverage reference transcriptome was generated from combined gill, lung and skin tissues of metamorphosing juveniles, and lung tissue of adults: 86,282 unigenes with total length of approximately 77,275,634 bp and N50 of 1732 bp were obtained. Among these, 13,246 unigenes were assigned to 288 pathways. To determine the possible involvement of miRNAs in the respiratory transition, small RNA libraries were sequenced; 282 miRNAs were identified, 65 among which were known and 217 novel. Based on the hierarchical clustering analysis, the twelve studied samples were classified into three major clusters using differentially expressed miRNAs. We have validated ten differentially expressed miRNAs and some of their related target genes using qPCR. These results largely corroborated the results of transcriptomic and miRNA analyses. Finally, an miRNA-gene-network was constructed. Among them, two miRNAs with target genes related to oxygen sensing were differentially expressed between gill and lung tissues. Three miRNAs were differentially expressed between the lungs of larvae and lungs of adults.

**Conclusions:**

This study provides the first large-scale miRNA expression profile overview during the respiration transition from gills to lungs in Chinese giant salamander. Five differentially expressed miRNAs and their target genes were identified among skin, gill and lung tissues. These results suggest that miRNA profiles in respiratory tissues play an important role in the regulation of respiratory transition.

**Electronic supplementary material:**

The online version of this article (10.1186/s12864-018-4662-5) contains supplementary material, which is available to authorized users.

## Background

The endangered Chinese giant salamander (*Andrias davidianus*, Cryptobranchidae family), endemic to China, is the world’s largest amphibian. It has been called a “living fossil”, and it is referred to locally as wawayu (baby fish) because its vocalization is somewhat resemblant of a baby’s cry [1]. The Chinese government has declared this species a class II protected species [2]. Its ability to switch from aquatic to aerial respiration and the associated morpho-functional adjustments [3, 4] further contribute to its status of a species of great interest to biologists. Studies of this species might provide key information about body adaptions (anatomical, physiological and molecular) that enable organisms to transition from water to land.

Piiper (1982) divided the transition from water to land into three sub-stages dependent on different organs: fish gills, amphibian gills, and lungs and skin. The features of respiration in transitional states showed that the adjustment for gas exchange style in different breathing models is related to the metabolic rate [5]. Burggren and Doyle (1986) reported that the transition from gill respiration to lung respiration during the development of bullfrog larvae is accompanied by a decline in the regulation of branchial ventilation frequency reflex and attendant increasing dependence on breathing air [6]. Based on the neurorespiratory pattern of gill and lung ventilation, physiologists found that spinal nerve II is crucial for lung ventilation [7]. However, these studies merely identified the differences among different respiration styles. In transitional forms, depending on both gill and lung ventilation, gill and lung breathing can be replaced or complemented by skin and or buccopharyngeal breathing [8]. This adaptation is necessary for the adaptive changes of respiratory properties of blood and ventilatory and circulatory flow rates [8]. Nitric oxide synthase (NOS)/nitric oxide (NO) levels in gills and lungs of *Protopterus annectens* during aestivation and arousal changed in a tissue-specific manner in parallel with organ readjustment [9]. However, despite numerous efforts, signal transduction mechanisms that underlie the cyclic branchial and pulmonary remodeling remain incompletely explained. Assunta et al. (2016) studied the water-land transition from the perspective of gene expression patterns; they used lungfish transcriptome sequence (which is a time-effective and economical method [10–13]) and phylogenetic relationship analysis [14]. They identified 226 concatenated protein sequences in all vertebrates and 59, 951 informative amino acid positions in both lungfish/tetrapod and the coelacanth/lungfish + tetrapod nodes. They also identified several proteins in the tetrapod lung which were not found in the actinopterygian fish (pulmonary surfactants A, C, D SFTPA, SFTPC, and SFTPD). Regarding the regulation pattern of the expression of these target mRNAs, microRNAs (miRNAs) play an important role by specifically binding to the 3’UTR of mRNA and (usually) promoting mRNA degradation [15]. miRNAs are endogenous ~ 22 nucleotide-long non-coding RNAs, which regulate a range of essential cellular and biological processes by targeting mRNA for cleavage or translational repression [16–23]. There are indications that miRNAs modulate pulmonary development and maintain lung homeostasis [24, 25]. Misexpression of some miRNAs can contribute towards neurodevelopmental disorders, which can also affect the lung ventilation [26].

With two key developmental stages, Chinese giant salamander is a typical amphibian, undergoing a transition from aquatic larvae to terrestrial adults, with concomitant transfer of respiration from gills to lungs prior to the metamorphosis [27]. In order to unveil the role of miRNAs in the adjustment of respiratory system to the transition from water to land, we have studied the expression of miRNAs and their corresponding target genes in metamorphosing larvae of the Chinese giant salamander. Real time quantitative polymerase chain reaction (qPCR) was used to study expression in gill, lung and skin tissues. We also assembled a high-coverage reference transcriptome, and determined the putative regulatory network of miRNAs associated with the studied morpho-functional adjustments. This study contributes to the understanding of the role of miRNA-mediated regulatory networks in the transition from water to land of Chinese giant salamander.

## Methods

### Sample collection and RNA isolation

**Fig. 1:**
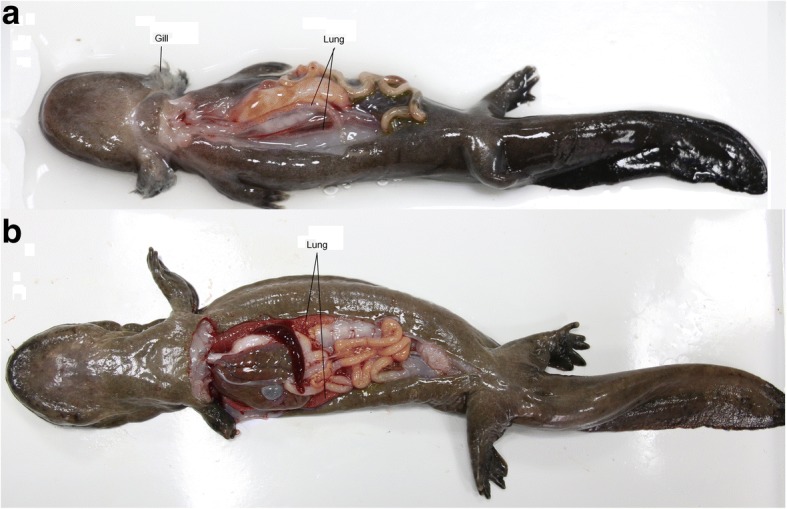
Chinese giant salamander respiratory system transition from gills, lungs and skin to respiration mainly using lungs (**a**) A transitioning Chinese giant salamander with gills and lungs; (**b**) Chinese giant salamander with lungs and without gills

Healthy Chinese giant salamander’s larvae (5 months-old) and adults (20 months-old) were obtained from the Jurong Chinese Giant Salamander Breeding Center in Zhenjiang, Jiangsu, China. Specimens were anaesthetized using 2-phenoxyethanol, euthanized using tricaine methanesulfonate (Sigma, USA), and promptly dissected. Three different tissues were collected from three larvae: skin (skin5m), gills (gill5m), and lungs (lungs5m); and only lungs from three adults (lungs20m) (Fig.1; Additional file 1: Table S1). This ensured triplicate samples for four different tissue groups (skin5m, gill5m, lungs5m, lungs20m). RNA was extracted from all samples using Trizol Reagent (Invitrogen, USA) according to the manufacturer’s instructions. Purity of RNA was checked by NanoPhotometer® spectrophotometer (IMPLEN, CA, USA). The final concentration was determined using Qubit® RNA Assay Kit in Qubit®2.0 Fluorometer (Life Technologies, CA, and USA). The integrity of RNA was assessed using the RNA Nano 6000 Assay Kit and Agilent Bioanalyzer 2100 system (Agilent Technologies, CA, and USA). The handling of animals was conducted in accordance with the guidelines for the care and use of animals for scientific purposes set by the Institutional Animal Care and Use Committee of the Freshwater Fisheries Research Center, Chinese Academy of Fishery Sciences, Wuxi, China.

### Library preparation and transcriptome sequencing

Sequencing libraries were constructed using 3 μg of RNA per sample using NEBNext®Ultra™ RNA Library Prep Kit for Illumina® (NEB, USA), following the manufacturer’s instructions. Index codes were added to attribute sequences to each sample. Briefly: mRNA was purified from total RNA using poly-T oligo-attached magnetic beads and fragmented using divalent cations under elevated temperature in NEBNext First Strand Synthesis Reaction Buffer (5X). First strand cDNA was transcribed using random hexamer primer and M-MuLV Reverse Transcriptase (RNase H). Second strand cDNA synthesis was then performed using DNA polymerase I and RNase H. Remaining overhangs were converted into blunt ends using exonuclease/polymerase. After adenylation of 3′ ends of DNA fragments, NEBNext adaptors with hairpin loop structure were ligated to prepare for hybridization. Library fragments were purified with AMPure XP system (Beckman Coulter, Beverly, USA) to select cDNA fragments with the length of 150~ 250 bp. In order to generate the products, we used 3 μl USER Enzyme (NEW ENGLAND BioLabs Inc., USA) with size-selected, adaptor-ligated cDNA at 37 °C for 15 min, followed by 5 min at 95 °C. PCR was performed with Phusion High-Fidelity DNA polymerase, universal PCR primers and index (X) primer. Ultimately, we purified PCR products with AMPure XP system and assessed the library quality on the Agilent Bioanalyzer 2100 system. We performed clustering of the index-coded samples on a cBot Cluster Generation System using TruSeq PE Cluster Kit v3-cBot-HS (Illumina) according to the manufacturer’s instructions. Finally, the libraries were sequenced using paired-end reads on an Illumina Hiseq 4000 platform.

### De novo transcriptome assembly and prediction of coding sequence

In order to obtain high-quality clean reads, the raw data were trimmed to remove reads containing adapter, reads containing ploy-N and low-quality reads. Q20, Q30, GC-content and sequence duplication level of the clean data were calculated. Clean data were then assembled de novo as described before [28]: the sequenced ‘left’ and ‘right’ files (read1 and read2 files respectively) from all samples were pooled into left.fq and right.fq files, respectively. Transcriptome assembly was finalized using these two large files, and the longest fragments per gene were designated as unigenes. All these operations were conducted using Trinity [29], with all parameters set to default.

### Gene function annotation, SSR detection and primer design

These unigenes were then queried against public databases for functional annotation: NCBI non-redundant protein database using BLASTX searches [30], Protein Families (Pfam) [31], orthologous groups of genes (EggNOG) [32] and Swiss-Prot database [33]. The cut-off of value for annotation in NR, Swissport, KEGG databases was set E to 1-e5 using blastx, whereas for the Pfam database the value was set E to 10 using hmmscan. In addition, the predicted CDS were classified into EggNOG categories using HMMER version 3.1 with an E-value cutoff of 1E-05 [34]. Kyoto Encyclopedia of Genes and Genomes database (KEGG) [35] was used to associate unigenes with metabolic pathways. Gene Ontology database (GO) [36] was used to classify the unigenes functionally into biological process, molecular functions and cellular components categories. GO term analysis was conducted by blast2go using the Nr annotation information. TransDecoder was used to predict the sequence of the Unigene coding region and its corresponding amino acid sequence, which was recommended by Trinity and Cuffinks. MIcroSAtellite identification tool (MISA) [37] was used to detect SSRs in the transcriptome with the following parameters: definition (unit size - min. repeats) = 1–10 2–6 3–5 4–5 5–5 6–5, and interruption (max. Difference between two SSRs) = 100. Six types of SSRs were investigated: mono-, di-, tri-, tetra-, penta-, and hexa-nucleotide repeats. Primers for SSRs were designed using Primer3 (http://bioinfo.ut.ee/primer3-0.4.0/).

### Quantification of gene expression levels

The read count for each gene was obtained by mapping clean reads to assembled transcriptome using RSEM [38]. Expression levels of unigenes were calculated as fragments per kb per million reads (FPKM). Differentially expressed genes (DEGs) were identified using DESeq R package (1.10.1) [39], employing a model based on the negative binomial distribution. Benjamini and Hochberg’s approach was used to adjust the resulting *P* value, which was determined by false discovery rate (FDR) and fold change (FC) [40]. In the pairwise analysis of the four groups (12 samples with 3 replicates) described before, any gene with an adjusted *P* < 0.05 and FC ≥ 2 was considered to be a differentially expressed gene (DEG).

### Library preparation and sRNA sequencing

A total amount of 1.5 μg RNA per sample was used as input material for the RNA sample preparations. Sequencing libraries were generated using NEBNext®Ultra™ small RNA Sample Library Prep Kit for Illumina® (NEB, USA) following manufacturer’s recommendations, and index codes were added to attribute sequences to each sample. First of all, 3’ SR Adaptor for Illumina was mixed with RNA and nuclease-free water. This was followed by incubation for 2 min at 70 °C in a preheated thermal cycler. Tube was then transferred to ice, and 3’ Ligation Reaction Buffer (2X) and 3’ Ligation Enzyme Mix. The mixture was incubated for 1 h at 25 °C in a thermal cycler. To prevent adaptor-dimer formation, the SR RT Primer hybridizes to the excess of 3′ SR Adaptor that has remained free after the 3′ ligation reaction, and transforms the single-stranded DNA adaptor into a double-stranded DNA molecule. After ligating the 5′ SR Adaptor, reverse transcription and PCR amplification were performed. At last, PCR products were purified (AMPure XP system) and library quality was assessed on the Agilent Bioanalyzer 2100 system. We performed the clustering of index-coded samples on a cBot Cluster Generation System using TruSeq PE Cluster Kit v4-cBot-HS (Illumina) according to the manufacturer′s instructions. After cluster generation, library preparations were sequenced on an Illumina HiSeq X Ten platform and single-end reads were generated.

### Small RNA annotation, new miRNAs, and prediction of their target genes

For small RNA sequencing reads, all sequences with adapter or poly-N contaminants and low-quality reads were flittered. After that, sequences < 15 and > 35 nt were removed. Q20, Q30, GC-content and sequence duplication level of the clean data were calculated as well. Using Bowtie software, the clean reads were aligned with Rfam, Silva, GtRNAdb and Repbase databases to filter the transfer RNAs (tRNA), small nuclear RNAs (snRNA), small nucleolar RNAs (snoRNA), ribosomal RNAs (rRNA) and other ncRNAs, as well as repeats. By comparing the remaining reads with known miRNAs from the miRBase, known miRNAs were detected and new miRNAs predicted. Non-conserved miRNAs were predicted using miRdeep2 [41] with the following run parameters: “-g -1 -b 0”. Secondary structure prediction of new miRNAs was performed using Randfold tools [42] with the parameters: s = simple mononucleotide shuffling, and number of randomizations = 99. As we did not have the whole genome sequence at disposal, RNA-seq data were used to predict new microRNAs.

### miRNA expression levels and functional annotation of their target genes

To estimate the miRNA expression levels for each sample, sRNAs were mapped back onto the precursor sequence [43]. From the mapping results we know the readcount, which represents the number of reads that are mapped to a certain miRNA. Then the expression of miRNA in each sample was normalized as transcripts per million (TPM): TPM = readcount× 1,000,000/Mapped Reads. Potential target genes of differentially expressed miRNAs were identified using miRnada v3.3a (run parameters: -sc 150.0 -en − 30 -scale 4.0 -go − 2.0 -ge − 8.0) and RNA hybrid v2.1.1 (run parameters: -m 50,000 -d 1.9,0.28 -b 1 -e − 30) programs. Intersection of the target set was regarded as the target gene of a microRNA. After these step, intersection of predicted targets were verified by target accessibility (PITA) [44].Target genes of miRNAs were subjected to BLASTX searches (E-value threshold of 1.00e-5) and annotated against the NCBI’s Nr and Pfam databases, as well as the EggNOG and Swiss-Prot databases again. Target genes were also subjected to GO analysis, and classified into biological process, cellular component and molecular function categories. Metabolic pathways were analyzed using the KEGG database.

### Validation of differentially expressed miRNAs and their corresponding target genes

Expression levels of the following randomly selected miRNAs were determined using predesigned TaqMan MicroRNA assay (Life Technologies, Foster City, CA, USA): aca-miR-142-5p, bta-miR-142-5p, gga-miR-34c-3p, aca-let-7a-5p, aca-miR-203-3p, aca-miR-203-5p, aja-miR-142, dre-miR-203a-5p, efu-miR-223 and ipu-miR-142. cDNA was synthesized from 100 ng of total RNA using TaqMan® MicroRNA Reverse Transcription kit (Life Technologies). qPCR was performed using 7900HT Fast Real-Time PCR System (Life Technologies), with primers listed in Additional file 1: Tables S2 and S3. Relative expression of miRNAs was determined using the 2-ΔΔCT method [45], with mamm *U6 small nuclear 1* (U6), which belongs to the class of small nuclear RNAs, used as an endogenous reference [46]. No significant differences in the *U6* expression were found among the four groups (Additional file 1: Tables S4). All reactions were performed in triplicate [37], data expressed as mean ± SE, and subjected to one-way analysis of variance (ANOVA) using SAS8.0 program.

## Results

### Assembly and annotation of *Andrias davidianus* transcriptome

To obtain a reference transcriptome for the study of microRNA regulation patterns during the transition from gill respiration to lung respiration, samples from individuals which only had lung tissue and from those possessing both gill and lung tissues at the same time were used for RNA-Seq analysis. Their mix was used to obtain the transcriptome sequence. A total of 41,878,756 bp of clean data were obtained after quality filtering (Table 1). As there are no available assembled and annotated genomic sequences of the Chinese giant salamander that could be used for transcriptome assembly, Trinity de novo assembler program was used to assemble all the trimmed reads. About 77.83% of clean reads were mapped to the newly assembled transcriptome. Finally, a total of 86,282 unigenes with total length of approximately 77,275,634 bp and N50 of 1732 bp were obtained (Table 1). These values are higher than the number of unigenes and N50 in previously reported Chinese giant salamander transcriptomes [47, 48]. Among these, 30,060 unigenes (34.84%) were < 300 bp in length, 33,789 (39.16%) were > 301 and < 1000 bp, and 22,433 (26.00%) were > 1000 bp (Additional file 2). The overall GC ratio of the unigenes was 49.19%. Most of the CDS were 200~ 300 bp in length (19,941, 31.01%), followed by 100~ 200 bp (15,984, 24%), and 300~ 400 bp (7652, 11.9%) (as predicted by Trans-Decoder). No CDS with length < 100 were found, but 1130 (1.76%) of CDS were > 3000 bp (Additional file 1: Table S5). A total of 13,199 SSRs were identified from 22,433 unigenes (> 1 kb) using MISA Perl script, with 816 (3.64%) unigenes containing more than one SSR. Among the different types of SSRs, mono-nucleotide repeats were the most abundant (10,332), followed by di-nucleotide (1233), tri-nucleotide (687), tetra-nucleotide (88) and penta-nucleotide repeats (4) (Additional file 1: Table S6). Frequency distribution of these putative cSSRs was also calculated (Additional file 3). On average, one cSSR locus was found for about every 5.9 kb of a unigene sequence.

**Table 1:** Assembly quality statistics of the Chinese giant salamander transcriptome

Parameter	Value
Raw reads	41,878,756
High quality bases	12,482,332,994
High quality reads % (≥Q30)	89.39%
Unigene number	86,282
Unigene total length	77,275,634
Average length (bp)	895.62
Unigene N50 length (bp)	1732

BLASTX and BLASTn searches against GO, KEGG, eggnog and Nr databases were conducted to validate and annotate the assembled unigenes; this produced 6390 (21.27%), 9236 (30.74%), 13,246 (44.09%), 16,180 (53.85%), 27,181 (90.48%), 28,247 (94.02%) unigene matches respectively (E-value ≤1.0E-5) (Additional file 1: Table S7). A set of 30,044 unigenes were annotated in these public databases via Trans-Decoder annotation. Functional annotation of all unigenes against protein databases showed that a total of 18,435 (61.36%) unigenes exhibit significant similarity to known proteins in the Pfam database, and a total of 13,341 (44.40%) in the Swissprot database. Four species with most BLASTX hits against the Nr protein database accounted for 42.86% of the identified unigenes: *Xenopus tropicalis* (14.16%), *Chrysemys picta* (13.89%), *Chelonia mydas* (7.76%) and *Latimeria chalumnae* (7.05%). These results are comparable to those reported in the larvae of tiger salamander and the amphibian species usually used for the respiratory transition studies - *Xenopus tropicalis* [49]. The remaining 57.14% were distributed as follows: *Pelodiscus annectens* (5.38%), *Xenopus laevis* (3.37%), *Anolis carolinensis* (3.22%), *Oncorhynchus mykiss* (3.16%), *Alligator mississippiensis* (2.90%), *Alligator sinensis* (2.82%), and other species (36.28%) (Additional file 4). In total, 9236 (10.70%) unigenes were allocated into three main GO categories (Additional file 5): within the cellular component category, cell (5172) and cell part (5171) terms were most prominent; within the molecular function category, GO terms were predominantly associated with binding activity (5022) and catalytic activity (3622); the biological process category was dominated by cellular process (5471) and single-organism process (5039). These results are almost identical to Huang et al.’s report [48] and similar to be found by Eo et al. [49]. In the the latter study [49] these terms were associated with transcripts differentially expressed between gills and lungs.

The unigenes were also queried (by BLASTX) in KEGG database to further explore their biological functions and interactions. A total of 13,246 unigenes were assigned to 288 pathways, among which ribosome (345), MAPK signaling pathway (311) and focal adhesion (303) were the largest three groups. In addition, 189 unigenes were associated with oxidative phosphorylation, 61 with glutathione metabolism, 46 with metabolism of xenobiotics by *cytochrome P450* and 45 with drug metabolism-cytochrome P450.

## Small RNA sequencing data and annotation

To assess the involvement of miRNAs in the respiratory transition, small RNA libraries were constructed for sequencing. A total of 183,422,095 clean reads were generated from 12 samples after removing low quality reads, adapter sequences, and sequences with lengths < 15 nt and > 35 nt (Additional file 1: Table S8). Q30 percentage per individual ranged from 98.83% to 99.13%. Reads from all libraries were annotated using GenBank and Rfam databases (Additional file 1: Table S9). By annotation, Rrnas, tRNAs, snRNAs and snoRNAs were separated from each other. tRNAs and rRNAs were the most abundant small RNA types. Length of mature miRNAs was mostly between 20 and 22 nt (27.65%), which were typical dicer-processed miRNA products (Additional file 6). The most abundant small RNAs in all of the libraries were 21 nt in length, and a significant bias towards U was observed in the first nucleotides of all putative miRNA sequences across all libraries (Additional file 7). U also had high frequency (51%) in positions 9 and 12. Moreover, A + U were dominant at the start position. These features might have effect on the miRNA target recognition [50]. To identify the conserved miRNAs in *A. davidianus*, small RNA sequences were compared with the currently available mature miRNAs in the miRBase. The miRNAs that perfectly matched with conserved miRNA sequences or had stem-loop precursors were identified as conserved miRNAs, which indicates their conserved function across various species. Finally, a total of 65 small RNAs in *A. davidianus* were identified as orthologs of known miRNAs in other species (Additional file 1: Table S10). For conserved miRNA, most of them ranged from 21 to 23 nt in length; sorted by abundance: 22 nt > 21 nt > 23 nt. These results are fully consistent with Yang et al.’s results in Chinese giant salamander [51]. Abundance of known miRNA family members ranged from 1 to 13. The most abundant were let-1 and mir-142 (13 and 12 members respectively). Among the remaining, two families had 9 members, and four families had > 3 members and < 9 members. To uncover novel miRNAs sequences*,* all non-annotated small RNAs were searched against the newly-assembled transcriptome. We identified 46 non-conserved miRNAs belonging to 32 miRNA families, five of which (families) contained more than one member. Length distribution of non-conserved miRNAs was identical to that of all miRNAs. Thus, length distribution of miRNAs might provide a window into the role of miRNAs components in morpho-functional adjustments, because length variation is related to enzymatic modifications, such as RNA editing and exonuclease actives [52].

### Differential expression of miRNAs in gills, lungs and skin

**Fig. 2:**
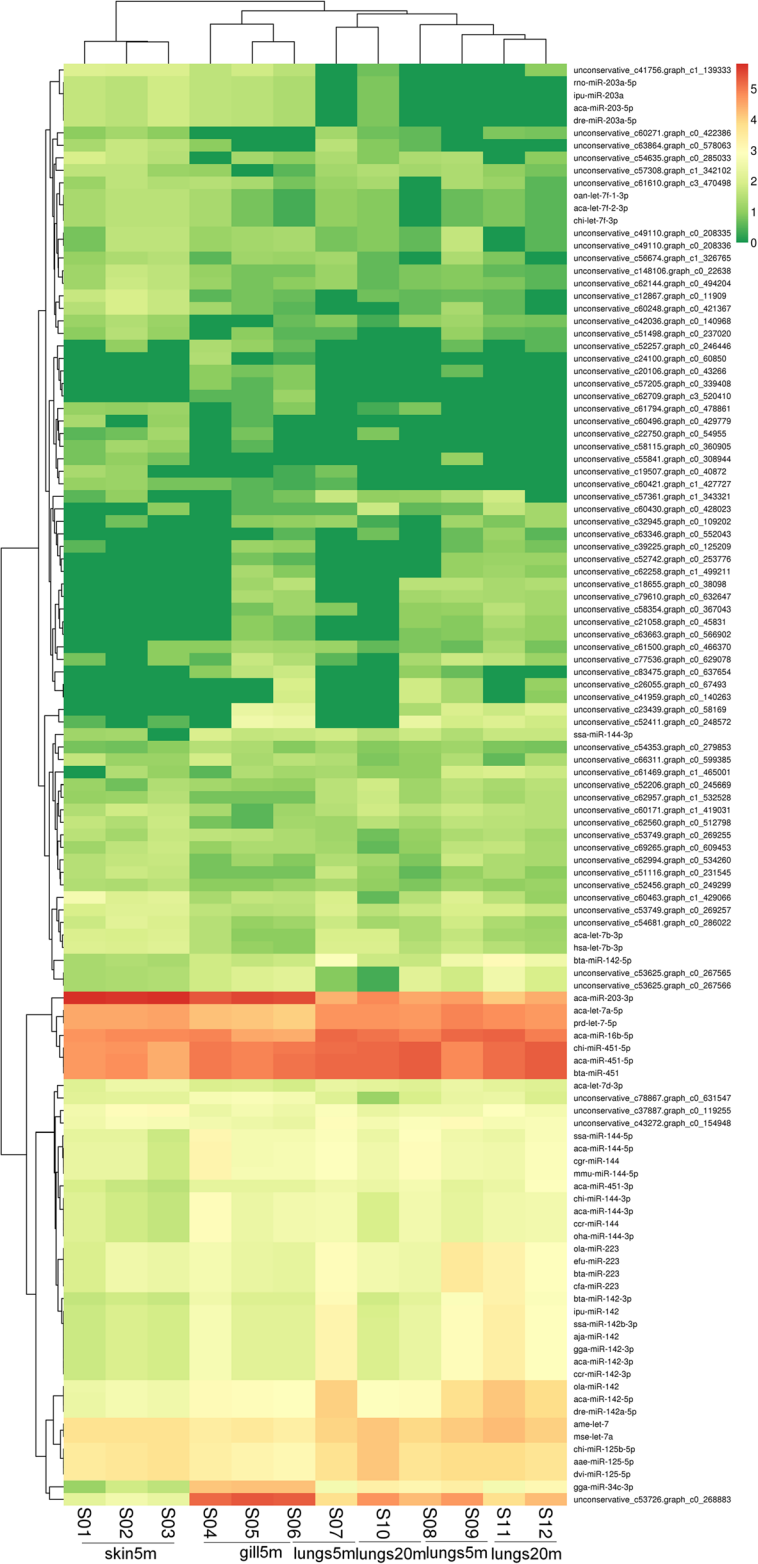
Hierarchical cluster analysis of differentially expressed miRNAs in the respiration tissue transfer process. Each row represents a different miRNA, and each column represents a different sample. Clustering based on parameter similarity is shown on the left (miRNAs) and above (samples) the figure. Increasing green intensity denotes decreasing gene expression, and increasing red intensity denotes increasing gene expression, with color scale shown on the right. The heatmap was constructed using expression profiling of each sample, with the data calculated from log10 (TPM + 1)

DESeq program was used to determine whether miRNA profiles were different between salamanders depending predominantly on gills and skin for respiration and those depending on lungs for respiration (Table 2). Between skin and gills in larvae, we found 77 differentially expressed miRNAs, 46 of which were up-regulated and 31 were down-regulated. Among them, four miRNAs were unique to skin and two to gills in larvae. All of these miRNAs belong to novel miRNAs. Between skin and lungs in larvae, we identified 69 differentially expressed miRNAs, 37 of which were up-regulated and 32 down–regulated. Among them, only one miRNA, named unconservative_c58354.graph_c0_367043, was unique to skin in larvae, and seven miRNAs were unique to gills in larvae. Between gills in larvae and lungs in adults, we identified 82 differentially expressed miRNAs, 55 up-regulated and 27 down–regulated. Among these, only one miRNA, named unconservative_c63346.graph_c0_552043, was unique to skin in larvae and three miRNAs were unique to gills in larvae. Between gills and lungs in larvae, we identified 32 differentially expressed miRNAs, half of which were up-regulated and half down–regulated. Here, six miRNAs were unique to lungs in larvae. Between gills in larvae and lungs in adults, we identified 45 differentially expressed miRNAs, 30 of which were up-regulated and 15 down–regulated. Among them, only one miRNA, named unconservative_c60271.graph_c0_422386, was unique to gills in larvae and four miRNAs were unique to lungs in adults. Between the adult and larva lungs, three miRNAs were differentially expressed including one up-regulated and two down-regulated. Results of all pairwise comparisons indicate that aca-miR-203-5p, dre-miR-203a-5p, ipu-miR-203a and rno-miR-203a-5p were expressed only in the lungs of larvae. Distribution of significant changes detected is shown as a volcano plot in the Additional file 8. According to the hierarchical clustering analysis, the 12 studied samples were classified into three major clusters, which corresponds to the three studied tissues (Fig. 2). Therefore, miRNA expression patterns indicate that miRNAs are involved in the regulation of adaptation of respiratory tissues during the transition from aquatic larvae to terrestrial adults.

**Table 2:** miRNA count among different tissues

Type	DEG Number	Up-regulated	Down-regulated
skin_5 m vs gills_5m	77	46	31
skin_5 m vs lungs_5m	69	37	32
skin_5 m vs lungs_20m	82	55	27
gills_5 m vs lungs_5m	32	16	16
gills_5 m vs lungs_20m	45	30	15
lungs_5 m vs lungs_20m	3	1	2

5 m and 20 m indicate the age of sampled specimens

**Fig. 3:**
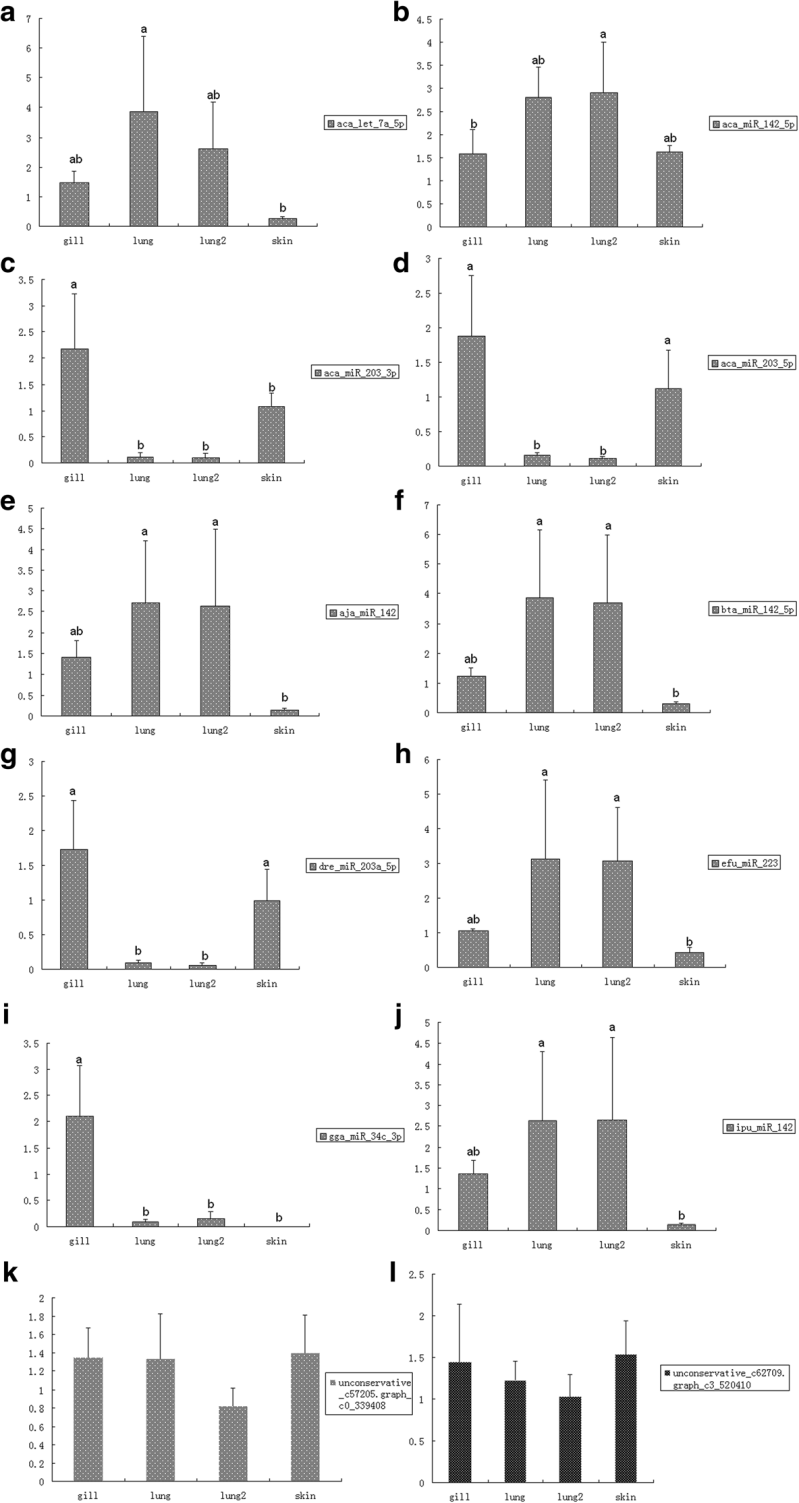
Validation of differentially expressed miRNAs in the respiration tissue transfer process. Different letters above bars indicate that two means are significantly different (*P* < 0.05)

Among the large number of differentially expressed miRNAs, we selected ten for further validation. These were chosen based on two criteria, their strong differential expression among the above four tissues (> 2-fold increase) and their strong correlation with ventilation features, either strong positive or strong negative: a) no expression in some tissues, while expression was observed in other tissue(s) in differential expression dataset (using DESeq); b) FDRs were lower than 0.05 for all pairwise comparisons. Based on these criteria, aca-miR-142-5p, bta-miR-142-5p, gga-miR-34c-3p, aca-let-7a-5p, aca-miR-203-3p, aca-miR-203-5p, aja-miR-142, dre-miR-203a-5p, efu-miR-223 and ipu-miR-142 were selected for further validation experiments (Fig. 3). These results largely corroborated the results of transcriptomic analysis.

### Identification of target genes of differentially expressed miRNAs and their functional annotation

**Fig. 4:**
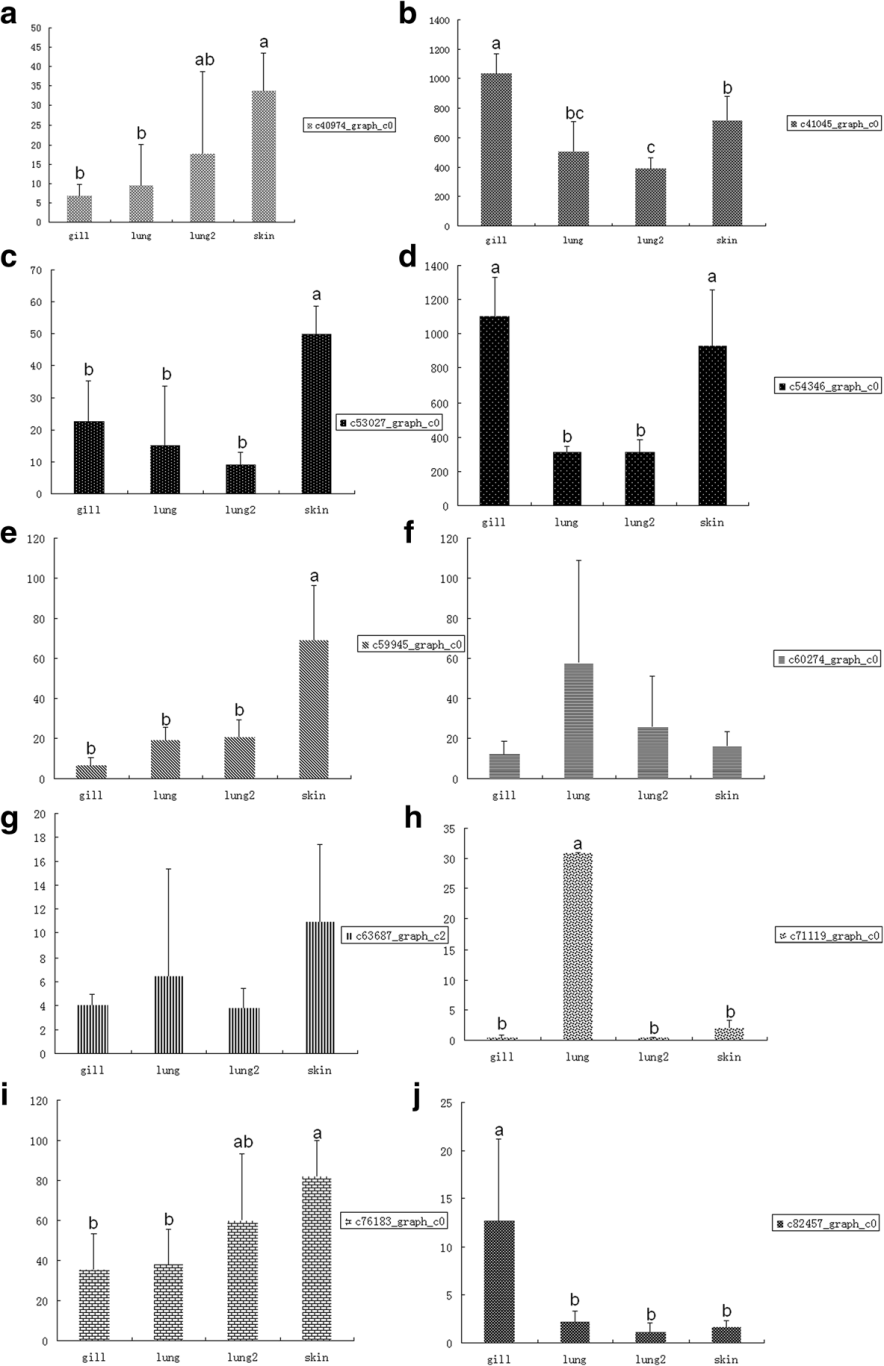
Validation of differentially expressed mRNAs in the respiration tissue transfer process. Different letters above bars indicate that two means are significantly different (*P* < 0.05). Note: c82457.graph_c0: sal-like protein 1; c54346.graph_c0: Semialdehyde dehydrogenase, NAD binding domain; c63687.graph_c2: sorting nexin-33; c53027.graph_c0: Ribosomal protein L11; c41045.graph_c0: Carbon-nitrogen hydrolase; c40974.graph_c0: Iron/zinc purple acid phosphatase-like protein C; c59945.graph_c0: glypican-5-like isoform X3; c60274.graph_c0: reverse transcriptase; c71119.graph_c0: centrosomal protein of 57 kDa isoform X1; c76183.graph_c0: trimethyllysine dioxygenase, mitochondrial isoform X3

The ultimate goal of our study was to test the hypothesis that miRNAs and their target genes are associated with the transition from aquatic to aerial respiration. Potential targets of differentially expressed miRNAs were identified by intersection of miRnada and RNA hybrid prediction programs. The target intersection set was verified by PITA program [44]. Among the 15,739 genes predicted as possible targets, 8358 (53.10%) were successfully annotated against the NR, Swiss-Prot, GO, KEGG and Pfam databases (Additional file 1: Table S11). A total of 352, 291, 1016, 117 and 862 differentially expressed miRNA target genes were found in pairwise comparisons of skin5 m vs gill5m, skin5 m vs lungs5m, skin5 m vs lungs20m, gill5 m vs lungs5 m and gill5 m vs lungs20m, respectively, but no differentially expressed genes were found in the lungs5 m vs lungs20m comparison. Expression levels of ten target genes related to the validated 12 miRNAs were also determined (Fig. 4). Among them, four genes had higher expression in skin, while two genes had higher expression in gills (both were compared to the lung tissue). One gene, *semialdehyde dehydrogenase*, had higher expression in both gills and skin, while only *centrosomal protein of 57 kDa isoform X1* exhibited higher expression level in lungs.

The top-scoring BLASTX hits against the Nr protein database indicated that the top four species were *Chrysemys picta* (17.12%), *Xenopus (Silurana)* (13.82%), *Chelonia mydas* (8.22%) and *Latimeria chalumnae* (7.69%), which accounted for nearly half of the miRNA targets identified against the Nr database. The remaining miRNA targets were distributed among *Pelodiscus sinensis* (4.81%), *Xenopus laevis* (4.55%), *Anolis carolinensis* (3.61%), *Alligator mississippiensis* (3.51%), *A. sinensis* (3.20%), *Python bivittatus* (1.68%), and other species (31.83%).

### Construction of the miRNA-gene-network

**Fig. 5:**
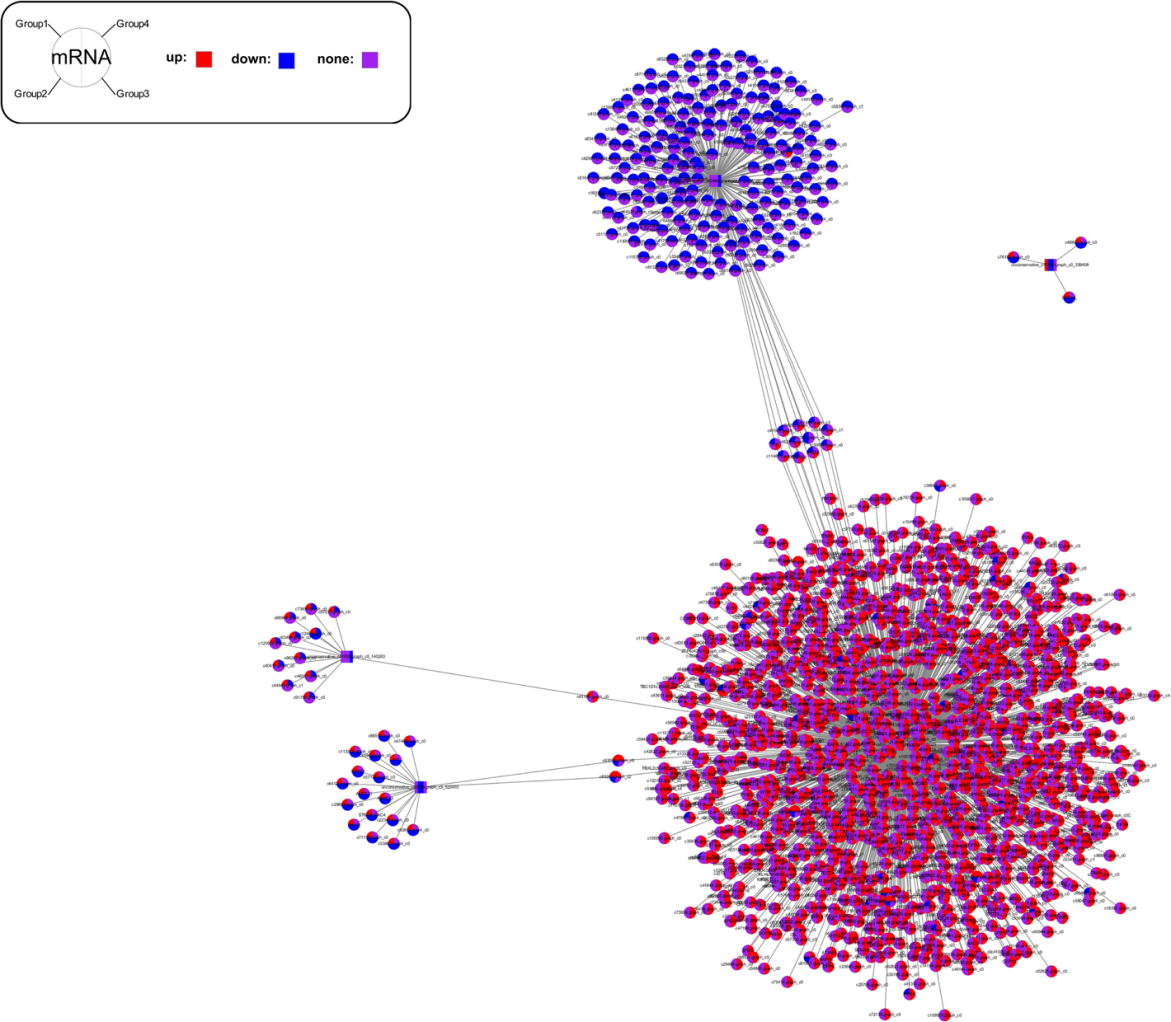
miRNA-Gene-Network for the respiratory transition in Chinese giant salamander. The miRNA-Gene-Network was built using gene expression data and predicted interactions in the TargetScan miRNA database. The squares represent miRNAs and the circles represent their target genes, where red indicates up-regulation, and blue - down-regulation, purple – no significant regulation. Group1 is skin5 m and Group2 is gill5m, Group3 is lungs5 m and Group4 is lungs20m. There are four pairwise group comparisons (skin5 m vs gill5m, gill5 m vs lungs5m, gill5 m vs lungs20m and lungs5 m vs lungs20m) for differential miRNA expression regulation pattern analysis. So different proportions of different colors in squares and cycles correspond to the differential expression levels among the four groups (pairwise comparisons); for example, if half of the square is filled with red, it means that this miRNA was up-regulated in two of the four groups

The ultimate goal of our study was to test the hypothesis that miRNAs and their target genes are associated with the transition from aquatic to aerial respiration. Thus, we identified five candidate miRNAs expressed in the specific expression patterns in gill, skin and lung. In order to identify putative functional modules, we constructed a miRNA-gene network (Fig. 5). Among the five miRNAs in the network, three were down-regulated during the respiratory transition: unconservative_c62709.graph_c3_520410, unconservative_c41959.graph_c0_140263 and unconservative_c60463.graph_c1_429066; whereas one exhibited increased expression during the respiratory transition: unconservative_c60430.graph_c0_428023. The miRNAs exhibiting the widest and narrowest degrees in the miRNA-Gene network were unconservative_c60430.graph_c0_428023 and unconservative_c57205.graph_c0_339408, which had 1361 and 3 target genes respectively. Regarding the total differentially expressed genes, most of them were targeted by two and more miRNAs in the network, whereas 18 genes were regulated by two miRNAs.

GO categorization of these network targets indicated a significant enrichment in the cellular process, single-organism process, metabolic process, biological regulation, cell, cell part, organelle, binding and catalytic activity (Additional file 9). These targets were also aligned to the KEGG database: a total of 203 differentially expressional miRNA target genes were involved in 185 pathways, with ‘focal adhesion’ and ‘MAPK signaling pathway’ being the most abundant terms (Additional file 10).

## Discussion

Our understanding of the mechanisms involved in the transition from gill respiration to lung respiration remains incomplete. In previous reports, candidate proteins [14], nitric oxide synthase (NOS)/nitric oxide [9], and conserved molecular networks [53] were reported to be involved in this complex process. Herein, we focused on the expression profiles of miRNAs. In order to associate the miRNAs with their target genes, a reference transcriptome of the Chinese giant salamander (*A. davidianus*) was generated. As a result, along with already assembled transcriptomes of the larval-stage tiger salamander [49] and Chinese salamander [54], scientists aiming to study the expression of genes in the transition from aquatic to aerial respiration now have transcriptomes of three salamander species at disposal.

In the present study, miRNA expression profiles were detected in three different tissues. Characteristics of the miRNAs detected, in particular 21 nt as the most common length and a significant bias towards U in the first nucleotide, are considered to be typical for miRNAs [55]. Based on these identified miRNAs, two steps were taken to better understand the association between miRNAs and their target genes during the transition from aquatic to aerial respiration.

Firstly, we focused on the specific expression of miRNAs in different tissues and different functions of these tissues during the respiration transition process. Expression profiles of gill and skin tissues could be distinguished from the expression profile of lung tissue using 115 miRNAs. Among these, we validated and confirmed the differential expression of 12 miRNAs. Among them, three new miRNAs differentially expressed between lungs of larvae and adults show specific expression patterns: unconservative_c60430.graph_c0_428023 was highly expressed in lungs of adults compared to lungs of larvae, while both unconservative_c41959.graph_c0_140263 and unconservative_c60463.graph_c1_429066 were highly expressed in lungs of larvae. Unconservative_c60430.graph_c0_428023 subsequently up-regulated the expressions of *secretory phospholipase A2 receptor isoform X2* gene, which is functionally associated with oxidative stress-induced premature senescence. Two down-regulated genes were *pulmonary surfactant-associated protein B precursor* and *pulmonary surfactant-associated protein B-like isoform X1*, which are necessary for vertebrate lung development [56]. These results are consistent with Eo et al.’s report [49], and illustrate that miRNAs contribute to the observed morpho-functional adjustments. In amphibians, at least 30% of oxygen exchange and most of the carbon dioxide elimination occurs through the skin [57]. The skin of lungless salamanders even serves as their only respiratory organ [58]. Among the five genes associated with respiration identified in Chinese giant salamander [27], *NADH-ubiquinone oxidoreductase chain 5*, *ATPase subunit 6* and *cytochrome b* were differentially expressed between skin and other tissues in the present study. Huang et al. [59] found that one of the most highly expressed genes related to the respiration in the skin of seven different anurans was *NADH-ubiquinone oxidoreductase* (GO term: “catalytic activity”), which is consistent with our result. Therefore, all of these results illustrate that gills, lungs and skin play an important role in the morpho-functional adjustments associated with respiration transition via oxidative stress, lung development and catalytic activity mechanisms.

Secondly, biological functions of miRNAs and their target genes involved in respiration regulation were classified. Five known miRNAs were related to hypoxia-induced respiration regulation. The *let-7* miRNA family members are induced by hypoxia [60], and increased level of *let-7a-5p* targets major pulmonary arterial hypertension pathways [61]. Higher expression level of *let-7a-5p*, and its target gene *sal-like protein 1* (*SALL1*), in gills in comparison to lungs illustrated that gill respiration might be a reflection hypoxia induced by the change of environment: from oxygen-abundant terrestrial to oxygen-scarce aquatic environment. Some studies have reported that sal-like protein 1 plays essential roles in maintaining self-renewal and pluripotency of embryonic stem cells (ESCs) [62] and promotes the intrahepatic cholangiocarcinoma cell migration [63]. These results indicate that cell differentiation and migration may be one possible way of promoting adjustments in the respiration process by sal-like protein 1. Other three miRNAs have been associated with the inhibition of hypoxia-induced adaptive changes: Wang et al. [64] reported that miR-142-3-P inhibits hypoxia-induced apoptosis and fibrosis of cardiomyocytes. For miR-142, Lu et al. [65] found that hypoxia-induced cell proliferation and invasion could be inhibited by the overexpression of miR-142 or hypoxia inducible factor1 (HIF-1α). Down-regulation of pulmonary miR-223 and upregulation of insulin-like growth factor 1 receptor expression can be initiated by pulmonary hypoxia in the right heart failure [66]. In our study, the three miRNAs mentioned above exhibited higher expression in lungs than in skin and gills, which indicates that pulmonary breathing is susceptible to adaptive air respiration by inhibiting the hypoxia-induced gene expression changes. However, miR-142 and miR-223 targets, *Ribosomal protein L11* and *Iron/zinc purple acid phosphatase-like protein C* respectively, had higher expression in skin. As they are involved in carbohydrate transport and metabolism, as well as ribosomal structure and biogenesis, this illustrates that metabolic ways were coordinated with adaptive changes in the skin respiratory patterns. For two miRNAs, unconservative_c57205.graph_c0_339408 and unconservative_c62709.graph_c3_520410, we did not observe a significant difference in expression among the studied tissues, but one of their targeted genes exhibited a significant difference in expression: *trimethyllysine dioxygenase* gene (validated by qPCR) had higher expression in skin compared to other tissues. As its mitochondrial isoform X3 is known to be down-regulated in response to hypoxia (10% O2) [67], this result illustrates that these two miRNAs facilitate the respiratory system transition from water to land by regulating the oxygen-related genes in lungs and skin. Eo et al. [49] used larval tiger salamander and pyrosequencing to identify genes differentially expressed between gill and lung tissues. They also found that ‘*pulmonary-associated protein isoform cra_a’* was exclusively expressed in the lungs. Among these differentially expressed miRNA-targeted genes, *Carbon-nitrogen hydrolase* (annotation in pFam) is associated with the “nitrogen compound metabolic process” GO term. This gene was involved in signal molecule changes in the gills and lungs of African lungfish during the maintenance and arousal phases of aestivation [9]. They also reported localization and expression of *nitric oxide synthase* (*NOS*), *Protein Kinase B*, *phospho-Akt* (*Akt*), *heat shock protein* 90 (*Hsp-90*), *hypoxia inducible factor α* (*HIF-1α*) in a tissue-specific manner in parallel with organ readjustment in the gills and lungs of *P. annectens* during aestivation and arousal [9]. Among these molecular components, qualitative and quantitative (immunofluorescence microscopy and Western blotting) patterns of *hypoxia inducible factor1* (*HIF1*) expression correlated inversely to that of nitric oxide synthase in both gills and lungs [9]. Synaptotagmin-like 4, associated with the GO terms “phospholipid binding” and “neurexin family protein binding”, is involved in intracellular trafficking, secretion, and vesicular transport. This gene indicates that lipid metabolism and nervous system may be related with the respiration transition. Zabelinskii et al. [68] studied the lipid and fatty acid composition of gills and lungs in 18 fish species and seven mammalian species, and found different molar ratios between phospholipids and cholesterol: 2:1 in fish and 3:1 in mammals. In vitro, cranial nerve (CN) roots and spinal nerve (SN) roots had different frequency, and their amplitude bursts patterns corresponded to gill and lung ventilatory burst patterns, respectively [69]. So, lipid metabolism and neural activity also play a key role in the process of morpho-functional adjustments.

## Conclusions

This study provides the first large-scale report of miRNA expression profiles and their predicted target genes during the respiration transition from gills to lungs in Chinese giant salamander. A high-coverage reference transcriptome was also generated and assembled. Two miRNAs with target genes related to oxygen sensing were differentially expressed between gill and lung tissues. Three miRNAs were differentially expressed between lungs of larvae and lungs of adults. Differential expressions of several miRNAs were validated and confirmed; e.g., *let-7a-5p* was targeting major pulmonary arterial hypertension pathways. Their target gene annotation indicates that the observed miRNA regulation pattern is a response to the change in the levels of oxygen in the environment. These results suggest that expression profiles of miRNAs in three respiratory tissues are correlated with oxygen responses during the transition from water to land.

## Additional files

Additional file 1: Supplementary **Tables S1** to **S11**. (DOC 122 kb)

Additional file 2: **Figure S1.** Size distribution of the assembled unigenes. Horizontal axis gives different size intervals of the assembled unigenes, vertical axis gives the number of unigenes located in the specific size interval. (PNG 44 kb)

Additional file 3: **Figure S2.** Frequency distribution of the putative cSSRs observed. C is the number of SSRs present in compound formation; C* is the number of sequences containing more than one SSR; p1 – p5 represent the numbers of mono-, di-, tri-, tetra- and penta-nucleotides respectively. (PNG 21 kb)

Additional file 4: **Figure S3.** Distribution of the top BLASTX hits for unigenes in the Nr databases. (PNG 64 kb)

Additional file 5: **Figure S4.** GO classification of Chinese giant salamander unigenes. 9236 (10.70%) unigenes were allocated into three main GO categories: biological process, cellular component and molecular function. Some of these unigenes were annotated with multiple GO terms. (PNG 109 kb)

Additional file 6: **Figure S5.** Length distribution of novel miRNAs. Horizontal axis gives the length of novel miRNAs, vertical axis gives the number of novel miRNAs. (PNG 110 kb)

Additional file 7: **Figure S6.** miRNA nucleotide bias. A: Nucleotide distribution at the first position of miRNA. Each row represents a miRNA of a different length, and each column represents the distribution of bases in the first nucleotide position. B: Overall nucleotide distribution. Each row represents a miRNA of a different length, and each column represents the overall distribution of the bases. (PNG 31 kb)

Additional file 8: **Figure S7.** Volcano plots of differentially expressed miRNA for different respiration-related tissues. Red dots represent significantly up-regulated genes and green dots significantly down-regulated genes in pairwise tissue comparisons (blue dots are genes with unchanged expression). The x-axis represents the log2-transformed gene expression. The y-axis is the *p* value (−log2) adjusted by Benjamini-Hochberg correction. All pairwise tissue comparisons revealed differentially expressed miRNAs. A: skin5 m vs gill5m; B: skin5 m vs lungs5m; C: skin5 m vs lungs20m; D: gill5 m vs lungs5m; E: gill5 m vs lungs20m; F: lungs5 m vs lungs20m. (PNG 742 kb)

Additional file 9: **Figure S8f.** Go classification of differentially expressed miRNA targets for the miRNA-gene-network. Differentially expressed miRNA targets were allocated into three main GO categories: biological process, cellular component and molecular function. Left vertical axis represents the percentage of genes, while right vertical axis indicates the number of genes. (PNG 1185 kb)

Additional file 10: **Figure S9f.** KEGG pathway analysis of differentially expressed miRNA targets for the miRNA-gene-network. The horizontal axis represents the number of genes, and the percentage of this number in the total number of genes in a given pathway. On the vertical axis are the names of KEGG pathways. (PNG 84 kb)

## Abbreviations

Akt: Phospho-Akt
CN: Cranial nerve
DEGs: Differentially expressed genes
EggNOG: Orthologous groups of genes
ESCs: Embryonic stem cells
FC: Fold change
FDR: False discovery rate
FPKM: Fragments per kb per million reads
gill5m: Larvae (5 months-old) gill
GO: Gene Ontology database
HIF-1α: Hypoxia inducible factor1
Hsp-90: Heat shock protein 90
KEGG: Kyoto Encyclopedia of Genes and Genomes database
lungs20m: Adults (20 months-old) lungs
lungs5m: Larvae (5 months-old) lungs
miRNAs: MicroRNAs
MISA: MIcroSAtellite identification tool
NO: Nitric oxide
NOS: Nitric oxide synthase
PITA: Target accessibility
qPCR: Real time quantitative polymerase chain reaction
RNase H: M-MuLV Reverse Transcriptase
rRNA: Ribosomal RNAs
SALL1: Sal-like protein 1
SFTPA, SFTPC, and SFTPD: Pulmonary surfactants A, C, D
skin5m: Larvae (5 months-old) skin
SN: Spinal nerve
snoRNA: Small nucleolar RNAs
snRNA: Small nuclear RNAs
SSR: Microsatellite
TPM: Transcripts per million
tRNA: Transfer RNAs
U6: mamm U6 small nuclear 1
