# Supplementary material for: Comparative expression analysis identifies the respiratory transition-related miRNAs and their target genes in tissues of metamorphosing Chinese giant salamander (Andrias davidianus)
Source: BMC Genomics. 2018 May 29;19:406. doi: 10.1186/s12864-018-4662-5 (PMC5975713; doi:10.1186/s12864-018-4662-5)
Supplement: Supplementary file 1 — Supplementary Tables S1 to S11. (DOC 122 kb) [file 12864_2018_4662_MOESM1_ESM.doc]

**Table S1**. Tissues sampled form Chinese giant salamander

| Item | Both lung and gill | Both lung and gill | | Only lung |
| --- | --- | --- | --- | --- |
| Tissue | Skin | Gill | Lung | Lung |
| Sample name | S01 | S04 | S07 | S10 |
| Sample name | S02 | S05 | S08 | S11 |
| Sample name | S03 | S06 | S09 | S12 |

**Table S2.** RT primers used for cDNA synthesis from miRNAs

| miRNA | RT primer |
| --- | --- |
| U6 | 5’ CGCTTCACGAATTTGCGTGTCAT 3’ |
| aca-miR-142-5p | 5’ GTCGTATCCAGTGCGTGTCGTGGAGTCGGCAATTGCACTGGATACGACTAGTAG 3’ |
| bta-miR-142-5p | 5’ GTCGTATCCAGTGCGTGTCGTGGAGTCGGCAATTGCACTGGATACGACGTAGTG 3’ |
| gga-miR-34c-3p | 5’ GTCGTATCCAGTGCGTGTCGTGGAGTCGGCAATTGCACTGGATACGACCCTGGC 3’ |
| aca-let-7a-5p | 5’ GTCGTATCCAGTGCGTGTCGTGGAGTCGGCAATTGCACTGGATACGACAACTAT 3’ |
| aca-miR-203-3p | 5’ GTCGTATCCAGTGCGTGTCGTGGAGTCGGCAATTGCACTGGATACGACCAAGTG 3’ |
| aca-miR-203-5p | 5’ GTCGTATCCAGTGCGTGTCGTGGAGTCGGCAATTGCACTGGATACGACCTGTTG 3’ |
| aja-miR-142 | 5’ GTCGTATCCAGTGCGTGTCGTGGAGTCGGCAATTGCACTGGATACGACTCCATA 3’ |
| dre-miR-203a-5p | 5’ GTCGTATCCAGTGCGTGTCGTGGAGTCGGCAATTGCACTGGATACGACACTGTT 3’ |
| efu-miR-223 | 5’ GTCGTATCCAGTGCGTGTCGTGGAGTCGGCAATTGCACTGGATACGACTTGGGG 3’ |
| ipu-miR-142 | 5’ GTCGTATCCAGTGCGTGTCGTGGAGTCGGCAATTGCACTGGATACGACTCCATA 3’ |

**Table S3.** Primers used for miRNA expression validation

| miRNA | Primer pair | Annealing temperature (℃) | Length (bp) |
| --- | --- | --- | --- |
| U6 | F: 5’ GCTTCGGCAGCACATATACTAAAAT 3’  R: 5’ CGCTTCACGAATTTGCGTGTCAT 3’ | 60 | 89 |
| aca-miR-142-5p | GSP:5' GGGGGCATAAAGTAGAAAGCA3'  R: 5' GTGCGTGTCGTGGAGTCG3' | 60 | 65 |
| bta-miR-142-5p | GSP:5' GGGGGGCATAAAGTAGAAAG3'  R: 5' GTGCGTGTCGTGGAGTCG3' | 60 | 64 |
| gga-miR-34c-3p | GSP:5' GGGGGAATCACTAACCACACA3'  R: 5' GTGCGTGTCGTGGAGTCG3' | 60 | 65 |
| aca-let-7a-5p | GSP:5' GGGGGTGAGGTAGAGGTTGT3'  R: 5' GTGCGTGTCGTGGAGTCG3' | 60 | 64 |
| aca-miR-203-3p | GSP:5' GGGGCGTGAAAGTTTAGGAC3'  R: 5' GTGCGTGTCGTGGAGTCG3' | 60 | 64 |
| aca-miR-203-5p | GSP:5'GGGGGAGTGGTTCTTAACAGTT3'  R: 5' GTGCGTGTCGTGGAGTCG3' | 60 | 64 |
| aja-miR-142 | GSP:5'GGGGGTGTAGTGTTTCCTACTT3'  R: 5' GTGCGTGTCGTGGAGTCG3' | 60 | 66 |
| dre-miR-203a-5p | GSP:5'GGGGAGTGGTTCTTAACAGTTC 3'  R: 5' GTGCGTGTCGTGGAGTCG3' | 60 | 66 |
| efu-miR-223 | GSP:5'GGGGGTGTCAGTTTGTCAAATA3'  R: 5' GTGCGTGTCGTGGAGTCG3' | 60 | 66 |
| ipu-miR-142 | GSP:5' GGGGGGTAGTGTTTCCTACTT3'  R: 5' GTGCGTGTCGTGGAGTCG3' | 60 | 65 |
| Unconservative_c57205.graph_c0_339408 | GSP:5'AGGGCAGCTGGATTGCAT3'  R: 5'GTGCGTGTCGTGGAGTCG3' | 60 | 62 |
| Unconservative_c62709.graph_c3_520410 | GSP:5' TTGGGGTGGACGGTGACT3'  R: 5' GTGCGTGTCGTGGAGTCG3' | 60 | 62 |

Note: GSP is the specific primer for targeted miRNA, R is a matched RT primer (see Table S2)

**Table S4**. U6 miRNA expression in different Chinese giant salamander samples

| Tissues | U6 Ct |
| --- | --- |
| Gill5m | 13.80±0.13 |
| Skin5m | 14.06±0.13 |
| Lungs5m | 13.69±0.13 |
| Lungs20m | 13.45±0.13 |

**Table S5.** Coding sequence length distribution in Unigenes

| Length_span | Number | % | Length_span | Number | % | Length_span | Number | % |
| --- | --- | --- | --- | --- | --- | --- | --- | --- |
| 0~100 | 0 | 0 | 1000~1100 | 1018 | 1.58 | 2000~2100 | 293 | 0.46 |
| 100~200 | 15984 | 24.86 | 1100~1200 | 879 | 1.37 | 2100~2200 | 277 | 0.43 |
| 200~300 | 19941 | 31.01 | 1200~1300 | 814 | 1.27 | 2200~2300 | 226 | 0.35 |
| 300~400 | 7652 | 11.9 | 1300~1400 | 669 | 1.04 | 2300~2400 | 228 | 0.35 |
| 400~500 | 3751 | 5.83 | 1400~1500 | 640 | 1 | 2400~2500 | 192 | 0.3 |
| 500~600 | 2351 | 3.66 | 1500~1600 | 569 | 0.88 | 2500~2600 | 163 | 0.25 |
| 600~700 | 1709 | 2.66 | 1600~1700 | 475 | 0.74 | 2600~2700 | 152 | 0.24 |
| 700~800 | 1387 | 2.16 | 1700~1800 | 414 | 0.64 | 2700~2800 | 125 | 0.19 |
| 800~900 | 1142 | 1.78 | 1800~1900 | 370 | 0.58 | 2800~2900 | 121 | 0.19 |
| 900~1000 | 1219 | 1.9 | 1900~2000 | 306 | 0.48 | 2900~3000 | 101 | 0.16 |
| >3000 | 1130 | 1.76 |  |  |  |  |  |  |

**Table S6.** Different types of SSRs in Unigenes

| SSR type | Number |
| --- | --- |
| c | 816 |
| c* | 39 |
| p1 | 10332 |
| p2 | 1233 |
| p3 | 687 |
| p4 | 88 |
| p5 | 4 |
| Total | 13199 |

Note: c: unigenes containing more than one SSR; C*: SSR present in compound formation contains cross; p1: mono-nucleotide repeats; p2: di-nucleotide repeats; p3: tri-nucleotide repeats; p4: tetra-nucleotide repeats; p5: penta-nucleotide repeats.

**Table. S7.** Annotation of Unigenes against GO, KEGG, eggNOG and Nr databases (BLASTX and BLASTn searches)

| Database | Number | 300≤length<1000 | length≥1000 |
| --- | --- | --- | --- |
| GO | 9236 | 2159 | 6328 |
| KEGG | 13246 | 3516 | 8269 |
| Pfam | 18435 | 4404 | 12327 |
| Swissprot | 13341 | 3258 | 9029 |
| eggNOG | 27181 | 8192 | 14852 |
| nr | 28247 | 8835 | 15262 |
| Total | 30044 | 9430 | 15461 |

**Table S8.** miRNA sequencing data statistics

| Sample | Raw reads | Low quality | Containing 'N' reads | Length<15 | Length>35 | Clean reads | Q30 (%) |
| --- | --- | --- | --- | --- | --- | --- | --- |
| S01 | 12207979 | 0 | 0 | 14867 | 0 | 12193112 | 99.06 |
| S02 | 13832484 | 0 | 0 | 18623 | 0 | 13813861 | 99 |
| S03 | 18280514 | 0 | 0 | 19864 | 0 | 18260650 | 99.12 |
| S04 | 10838734 | 0 | 0 | 11873 | 0 | 10826861 | 98.83 |
| S05 | 16159580 | 0 | 51 | 473580 | 533162 | 15152787 | 98.89 |
| S06 | 20246448 | 0 | 51 | 915953 | 471607 | 18858837 | 98.96 |
| S07 | 13821849 | 0 | 0 | 15965 | 0 | 13805884 | 99.09 |
| S08 | 16274140 | 0 | 64 | 332404 | 675485 | 15266187 | 98.94 |
| S09 | 18315200 | 0 | 46 | 276022 | 451676 | 17587456 | 98.89 |
| S10 | 16014619 | 0 | 0 | 7764 | 0 | 16006855 | 99.13 |
| S11 | 18725268 | 0 | 58 | 527641 | 551423 | 17646146 | 98.90 |
| S12 | 14741475 | 0 | 47 | 323876 | 414093 | 14003459 | 98.85 |

**Table S9.** Structural annotation of sRNAs in different samples

| Sample | Total | rRNA | scRNA | snRNA | snoRNA | tRNA | Repbase | unannotated |
| --- | --- | --- | --- | --- | --- | --- | --- | --- |
| S01 | 12193112 | 2478564 | 0 | 4 | 2554 | 141816 | 43902 | 9526272 |
| S02 | 13813861 | 4090156 | 0 | 4 | 2902 | 166379 | 35983 | 9518437 |
| S03 | 18260650 | 4180392 | 0 | 2 | 4170 | 188640 | 49514 | 13837932 |
| S04 | 10826861 | 1538173 | 0 | 0 | 1226 | 2283734 | 18005 | 6985723 |
| S05 | 15152787 | 1751763 | 0 | 10 | 2912 | 4721660 | 73555 | 8602887 |
| S06 | 18858837 | 1874875 | 0 | 12 | 4651 | 5083158 | 125724 | 11770417 |
| S07 | 13805884 | 2446333 | 0 | 4 | 3364 | 583964 | 72759 | 10699460 |
| S08 | 15266187 | 1941557 | 0 | 7 | 2920 | 5194150 | 61853 | 8065700 |
| S09 | 17587456 | 1057017 | 0 | 5 | 2974 | 4949075 | 59087 | 11519298 |
| S10 | 16006855 | 1169934 | 0 | 1 | 3163 | 122736 | 70266 | 14640755 |
| S11 | 17646146 | 1262798 | 0 | 9 | 4186 | 5502794 | 63454 | 10812905 |
| S12 | 14006459 | 812966 | 0 | 7 | 2855 | 5023898 | 57546 | 8106187 |

Total: total number of clean Reads; rRNA: number of rRNAs; scRNA: number of scRNAs; snRNA: number of snRNAs; snoRNA: number of snoRNAs; tRNA: number of tRNAs; Repbase: number of repeated sequence reads; Unannotated: unannotated reads

**Table S10.** Identified and predicted miRNAs

| Samples | Known-miRNAs | Novel-miRNAs | Total |
| --- | --- | --- | --- |
| S01 | 64 | 153 | 217 |
| S02 | 64 | 143 | 207 |
| S03 | 62 | 158 | 220 |
| S04 | 62 | 138 | 200 |
| S05 | 60 | 168 | 228 |
| S06 | 60 | 192 | 252 |
| S07 | 60 | 162 | 222 |
| S08 | 51 | 156 | 207 |
| S09 | 59 | 168 | 227 |
| S10 | 60 | 132 | 192 |
| S11 | 57 | 168 | 225 |
| S12 | 60 | 154 | 214 |
| Total | 65 | 217 | 282 |

**Table S11.** Statistics of miRNA target annotation results in six different databases

| Type | GO | KEGG | Pfam | Swissprot | eggNOG | nr |
| --- | --- | --- | --- | --- | --- | --- |
| S01_S02_S03_vs_S04_S05_S06 | 400 | 544 | 810 | 599 | 960 | 991 |
| S01_S02_S03_vs_S07_S08_S09 | 406 | 531 | 809 | 602 | 939 | 968 |
| S01_S02_S03_vs_S10_S11_S12 | 596 | 804 | 1179 | 877 | 1440 | 1488 |
| S04_S05_S06_vs_S07_S08_S09 | 147 | 197 | 297 | 220 | 357 | 362 |
| S04_S05_S06_vs_S10_S11_S12 | 423 | 556 | 810 | 603 | 1008 | 1035 |
| S07_S08_S09_vs_S10_S11_S12 | 322 | 421 | 607 | 450 | 763 | 787 |
