# Supplementary figures and images for: Comparative expression analysis identifies the respiratory transition-related miRNAs and their target genes in tissues of metamorphosing Chinese giant salamander (Andrias davidianus)
Source: BMC Genomics. 2018 May 29;19:406. doi: 10.1186/s12864-018-4662-5 (PMC5975713; doi:10.1186/s12864-018-4662-5)

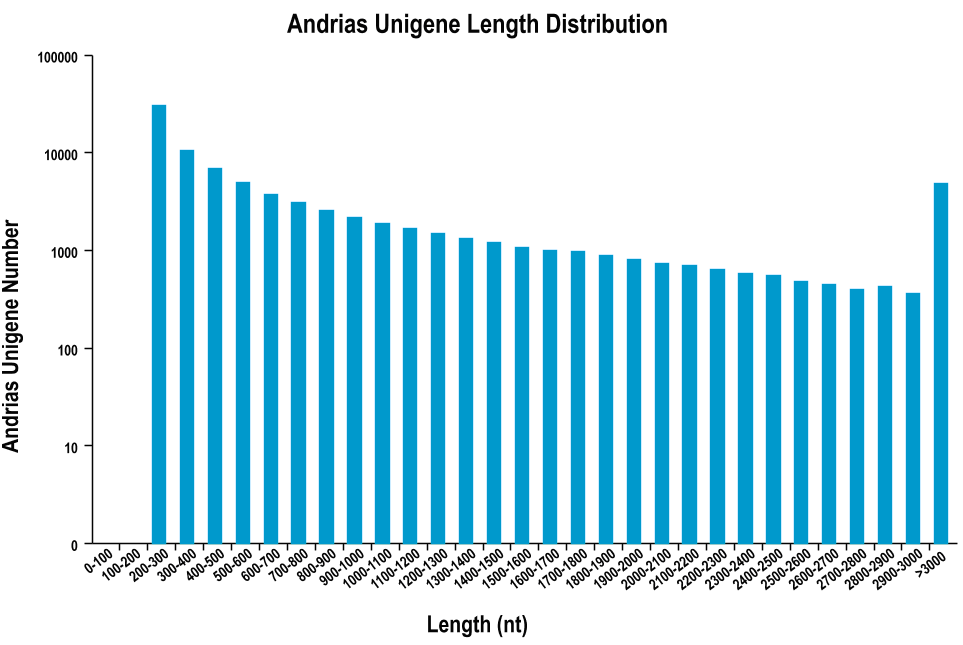

Supplement: Supplementary file 2 — Figure S1. Size distribution of the assembled unigenes. Horizontal axis gives different size intervals of the assembled unigenes, vertical axis gives the number of unigenes located in the specific size interval. (PNG 44 kb) [file 12864_2018_4662_MOESM2_ESM.png]

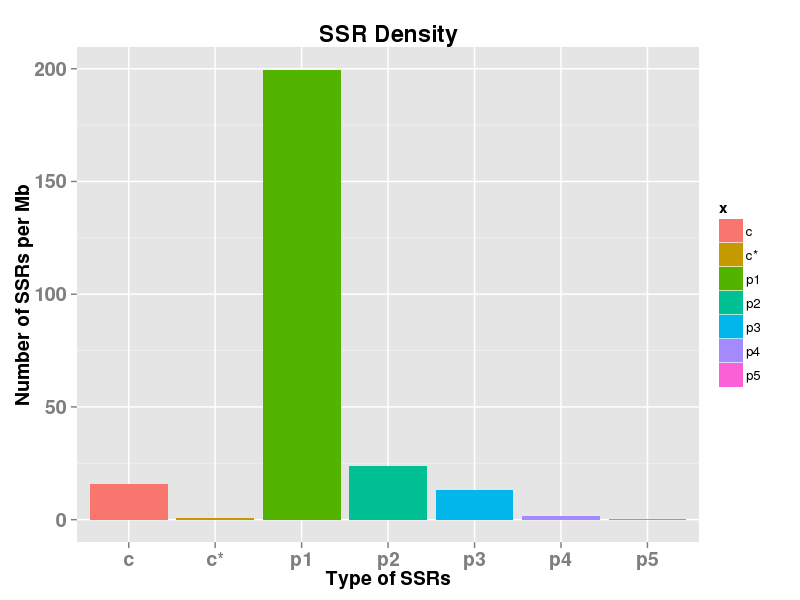

Supplement: Supplementary file 3 — Figure S2. Frequency distribution of the putative cSSRs observed. C is the number of SSRs present in compound formation; C* is the number of sequences containing more than one SSR; p1 – p5 represent the numbers of mono-, di-, tri-, tetra- and penta-nucleotides respectively. (PNG 21 kb) [file 12864_2018_4662_MOESM3_ESM.png]

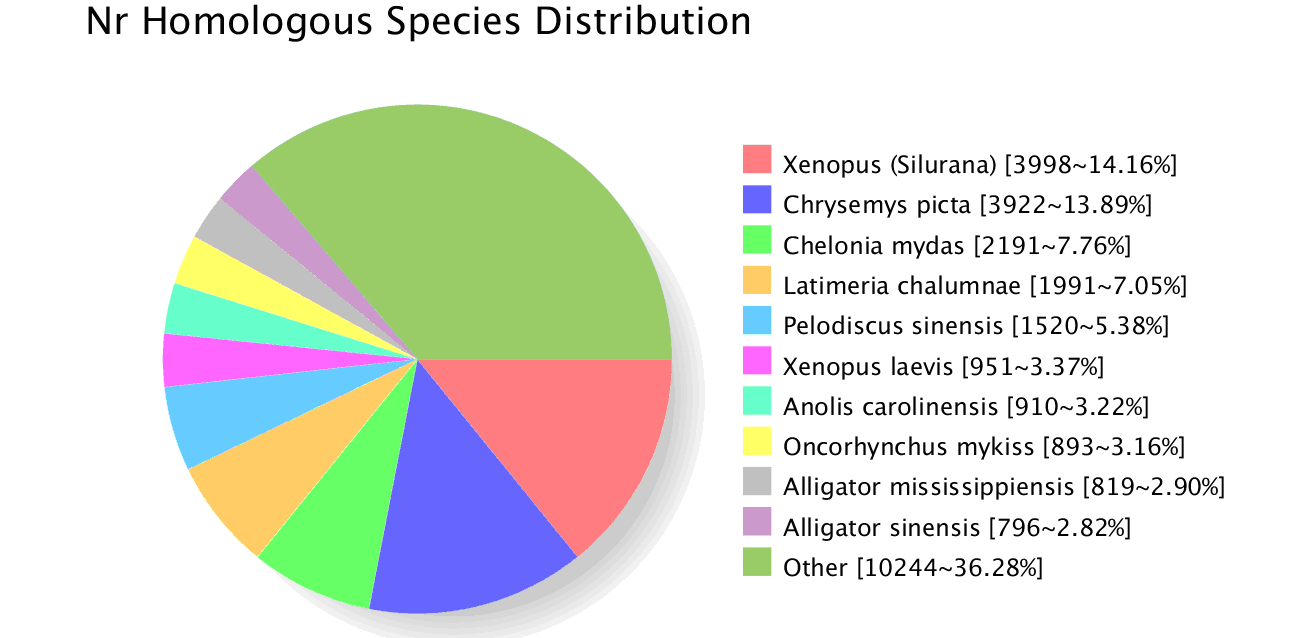

Supplement: Supplementary file 4 — Figure S3. Distribution of the top BLASTX hits for unigenes in the Nr databases. (PNG 64 kb) [file 12864_2018_4662_MOESM4_ESM.png]

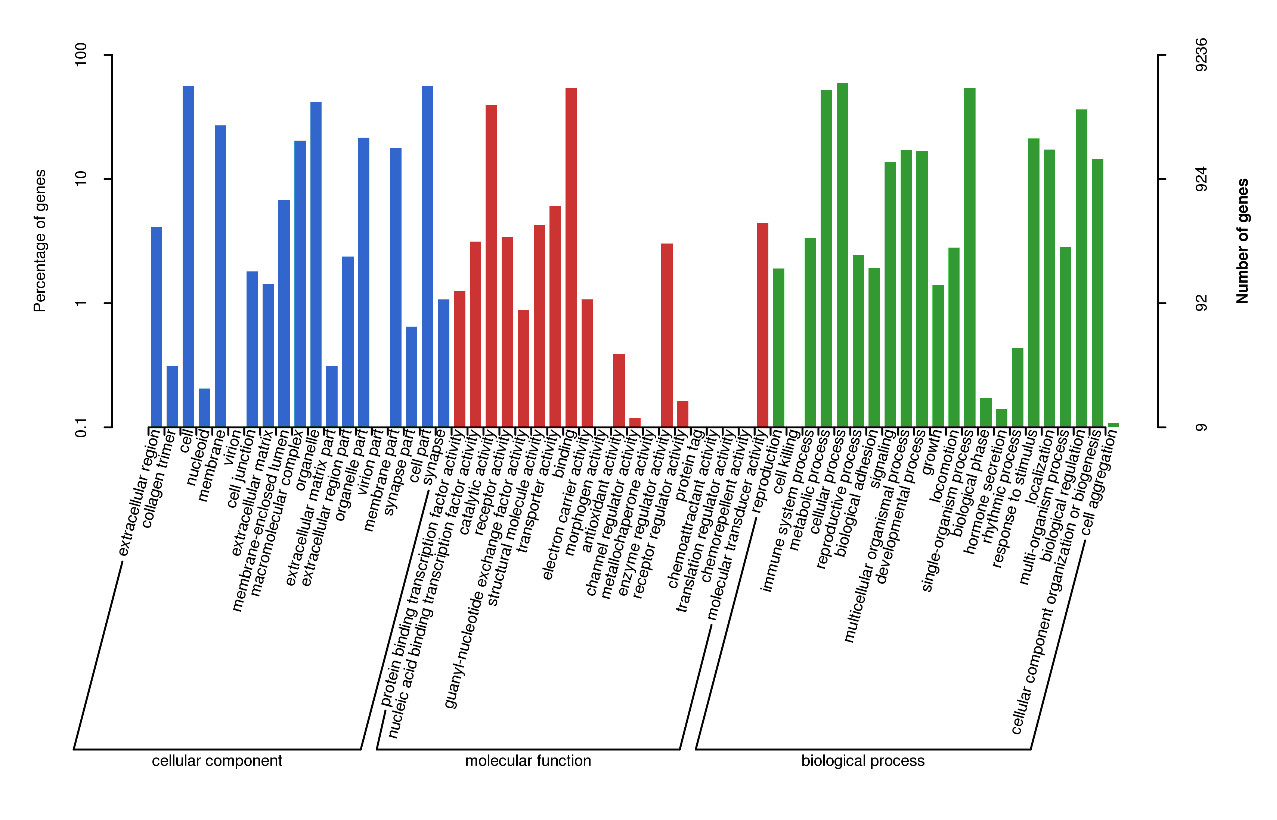

Supplement: Supplementary file 5 — Figure S4. GO classification of Chinese giant salamander unigenes. 9236 (10.70%) unigenes were allocated into three main GO categories: biological process, cellular component and molecular function. Some of these unigenes were annotated with multiple GO terms. (PNG 109 kb) [file 12864_2018_4662_MOESM5_ESM.png]

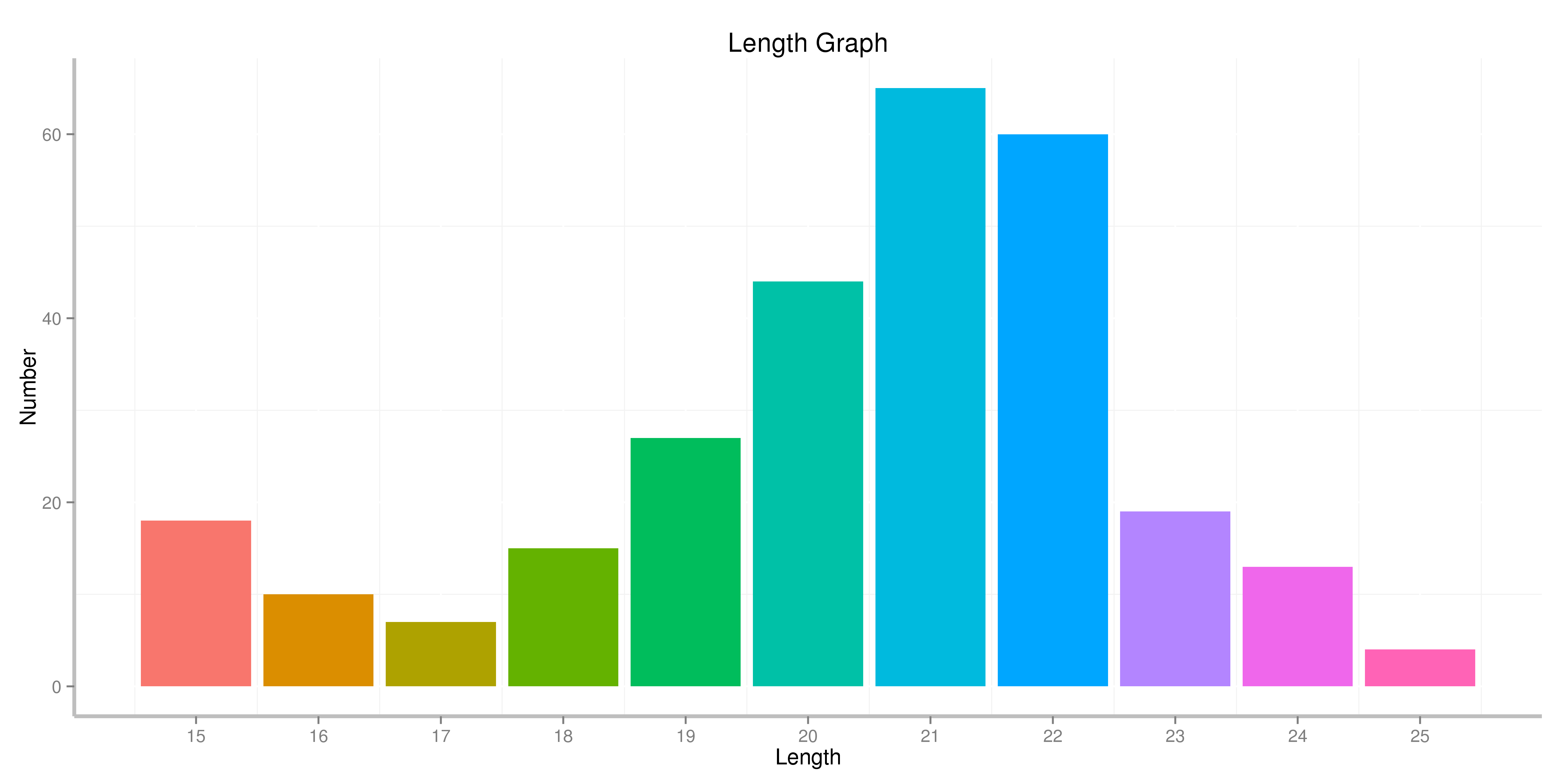

Supplement: Supplementary file 6 — Figure S5. Length distribution of novel miRNAs. Horizontal axis gives the length of novel miRNAs, vertical axis gives the number of novel miRNAs. (PNG 110 kb) [file 12864_2018_4662_MOESM6_ESM.png]

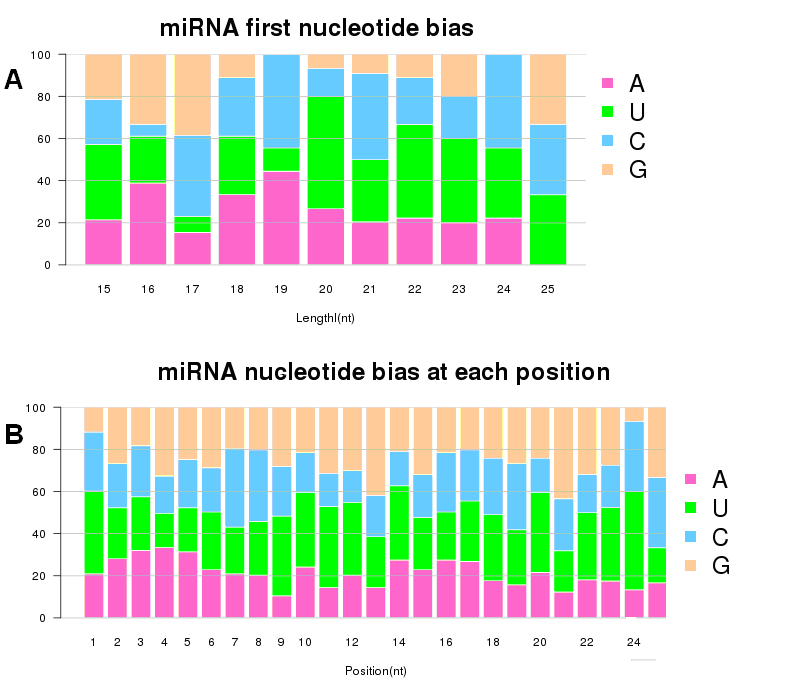

Supplement: Supplementary file 7 — Figure S6. miRNA nucleotide bias. A: Nucleotide distribution at the first position of miRNA. Each row represents a miRNA of a different length, and each column represents the distribution of bases in the first nucleotide position. B: Overall nucleotide distribution. Each row represents a miRNA of a different length, and each column represents the overall distribution of the bases. (PNG 31 kb) [file 12864_2018_4662_MOESM7_ESM.png]

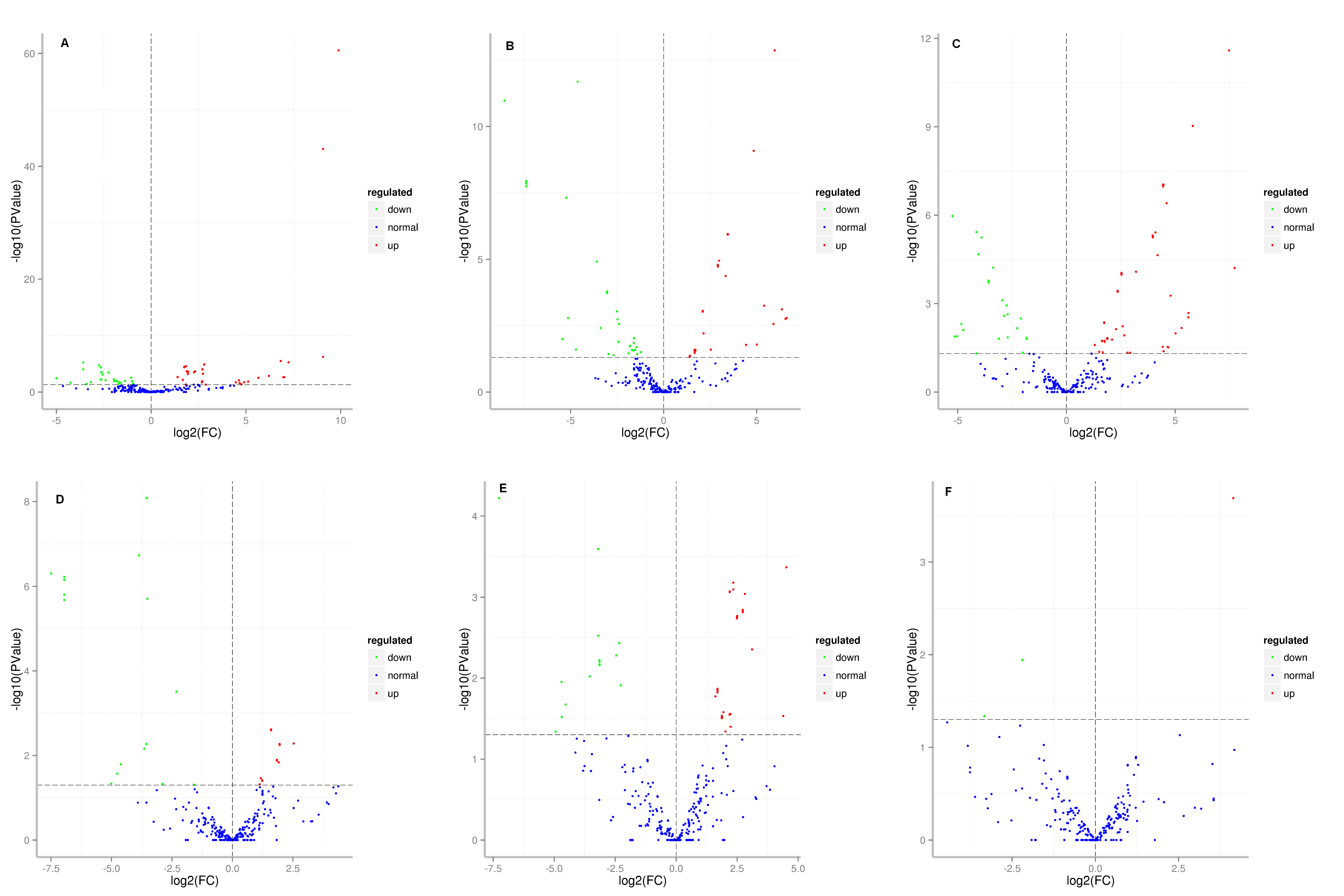

Supplement: Supplementary file 8 — Figure S7. Volcano plots of differentially expressed miRNA for different respiration-related tissues. Red dots represent significantly up-regulated genes and green dots significantly down-regulated genes in pairwise tissue comparisons (blue dots are genes with unchanged expression). The x-axis represents the log2-transformed gene expression. The y-axis is the p value (−log2) adjusted by Benjamini-Hochberg correction. All pairwise tissue comparisons revealed differentially expressed miRNAs. A: skin5 m vs gill5m; B: skin5 m vs lungs5m; C: skin5 m vs lungs20m; D: gill5 m vs lungs5m; E: gill5 m vs lungs20m; F: lungs5 m vs lungs20m. (PNG 742 kb) [file 12864_2018_4662_MOESM8_ESM.png]

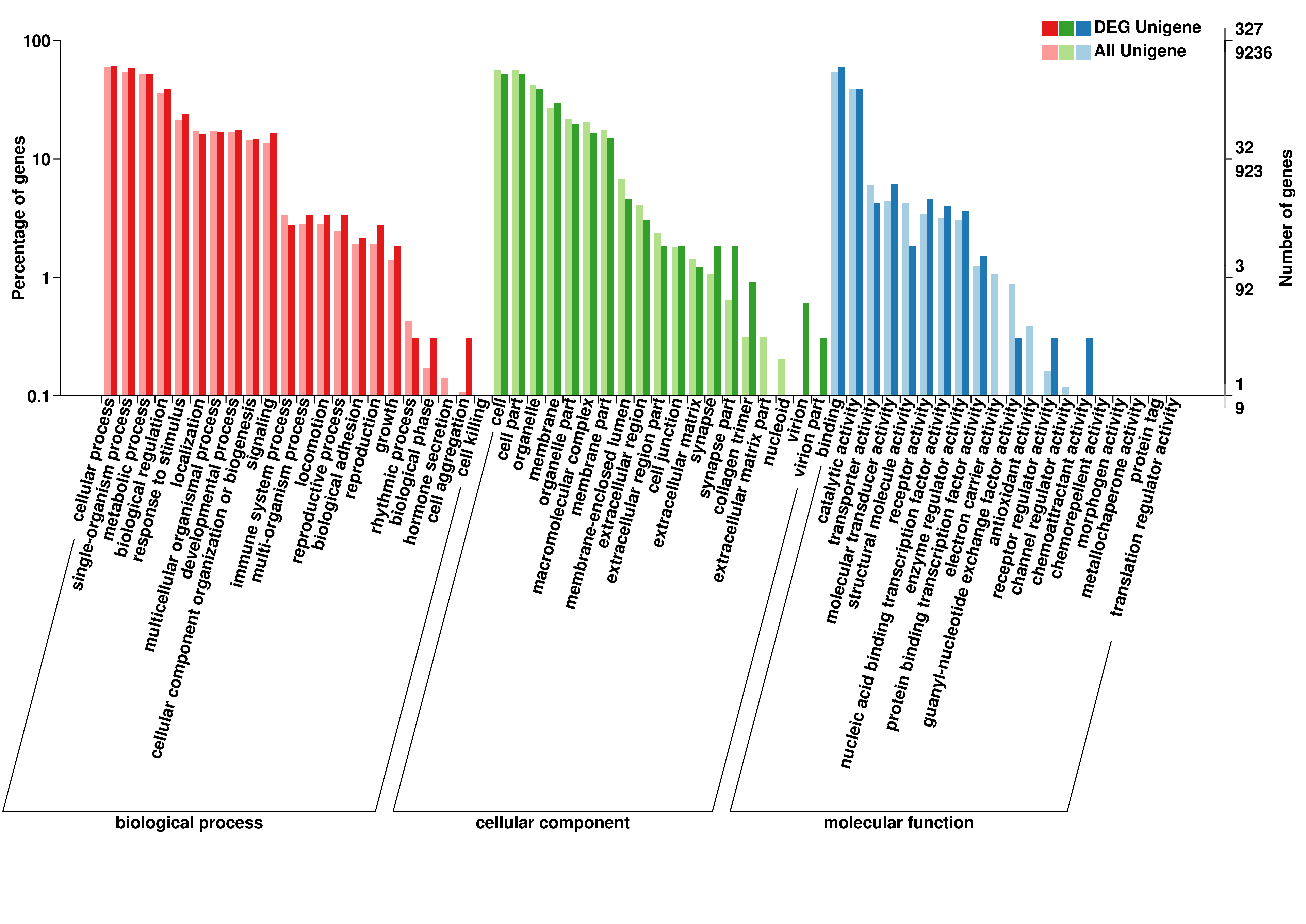

Supplement: Supplementary file 9 — Figure S8f. Go classification of differentially expressed miRNA targets for the miRNA-gene-network. Differentially expressed miRNA targets were allocated into three main GO categories: biological process, cellular component and molecular function. Left vertical axis represents the percentage of genes, while right vertical axis indicates the number of genes. (PNG 1185 kb) [file 12864_2018_4662_MOESM9_ESM.png]

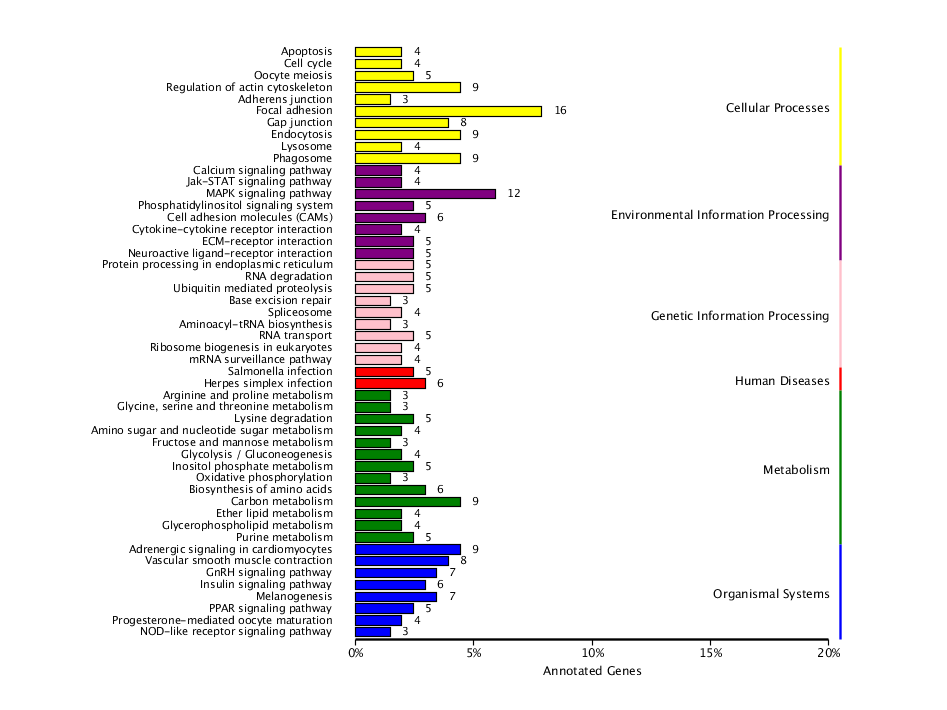

Supplement: Supplementary file 10 — Figure S9f. KEGG pathway analysis of differentially expressed miRNA targets for the miRNA-gene-network. The horizontal axis represents the number of genes, and the percentage of this number in the total number of genes in a given pathway. On the vertical axis are the names of KEGG pathways. (PNG 84 kb) [file 12864_2018_4662_MOESM10_ESM.png]
